# Exploring Successful Cognitive Aging: Insights Regarding Brain Structure, Function, and Demographics

**DOI:** 10.3390/brainsci13121651

**Published:** 2023-11-29

**Authors:** Xinze Xu, Lan Lin, Shuicai Wu, Shen Sun

**Affiliations:** 1 Department of Biomedical Engineering, Faculty of Environment and Life, Beijing University of Technology, Beijing 100124, China; xuxinze@emails.bjut.edu.cn (X.X.); wushuicai@bjut.edu.cn (S.W.); sunshen@bjut.edu.cn (S.S.); 2 Intelligent Physiological Measurement and Clinical Translation, Beijing International Base for Scientific and Technological Cooperation, Beijing University of Technology, Beijing 100124, China

**Keywords:** cognitive aging, MRI, cognitive resilience, neuroimaging, multiparametric imaging

## Abstract

In the realm of cognitive science, the phenomenon of “successful cognitive aging” stands as a hallmark of individuals who exhibit cognitive abilities surpassing those of their age-matched counterparts. However, it is paramount to underscore a significant gap in the current research, which is marked by a paucity of comprehensive inquiries that deploy substantial sample sizes to methodically investigate the cerebral biomarkers and contributory elements underpinning this cognitive success. It is within this context that our present study emerges, harnessing data derived from the UK Biobank. In this study, a highly selective cohort of 1060 individuals aged 65 and above was meticulously curated from a larger pool of 17,072 subjects. The selection process was guided by their striking cognitive resilience, ascertained via rigorous evaluation encompassing both generic and specific cognitive assessments, compared to their peers within the same age stratum. Notably, the cognitive abilities of the chosen participants closely aligned with the cognitive acumen commonly observed in middle-aged individuals. Our study leveraged a comprehensive array of neuroimaging-derived metrics, obtained from three Tesla MRI scans (T1-weighted images, dMRI, and resting-state fMRI). The metrics included image-derived phenotypes (IDPs) that addressed grey matter morphology, the strength of brain network connectivity, and the microstructural attributes of white matter. Statistical analyses were performed employing ANOVA, Mann–Whitney U tests, and chi-square tests to evaluate the distinctive aspects of IDPs pertinent to the domain of successful cognitive aging. Furthermore, these analyses aimed to elucidate lifestyle practices that potentially underpin the maintenance of cognitive acumen throughout the aging process. Our findings unveiled a robust and compelling association between heightened cognitive aptitude and the integrity of white matter structures within the brain. Furthermore, individuals who exhibited successful cognitive aging demonstrated markedly enhanced activity in the cerebral regions responsible for auditory perception, voluntary motor control, memory retention, and emotional regulation. These advantageous cognitive attributes were mirrored in the health-related lifestyle choices of the surveyed cohort, characterized by elevated educational attainment, a lower incidence of smoking, and a penchant for moderate alcohol consumption. Moreover, they displayed superior grip strength and enhanced walking speeds. Collectively, these findings furnish valuable insights into the multifaceted determinants of successful cognitive aging, encompassing both neurobiological constituents and lifestyle practices. Such comprehensive comprehension significantly contributes to the broader discourse on aging, thereby establishing a solid foundation for the formulation of targeted interventions aimed at fostering cognitive well-being among aging populations.

## 1. Introduction

Successful aging, in accordance with the criteria established by Rowe and Kahn [1], refers to the achievement of a high level of physical, psychological, and social functioning as individuals enter old age. In a comprehensive meta-analysis conducted by Kim and Park [2], four defining domains emerged, encapsulating the factors associated with successful aging. These domains encompass the avoidance of disease and disability, the maintenance of high levels of cognitive, mental, and physical functioning, active participation in various aspects of life, and the achievement of psychological well-being during later stages of life. The achievement of successful aging is intricately interwoven with the capacity to sustain a certain level of cognitive prowess and effectively navigate one’s environment throughout the aging trajectory. However, it is imperative to acknowledge the substantial variations witnessed in cognitive capacities among older adults, as underscored by Zhang et al. [3]. Rowe and Kahn [4] emphasize that cognitively successful older adults exhibit distinctive profiles across various cognitive domains. The traditional dichotomy of normal versus pathological disparities proves insufficient in providing a comprehensive depiction of the intricate nature of cognitive aging. Hence, they have further partitioned the domain of normal cognitive aging into three discernible categories: accelerated cognitive aging, normal cognitive aging, and successful cognitive aging. Within this framework, successful cognitive aging encompasses not only the absence of cognitive impairment but also the ability to excel in multidimensional cognitive assessments [5].

Individuals characterized by successful cognitive aging represent a distinct cohort distinguished by cognitive performance exceeding that of age- and education-matched older adults [6,7]. Remarkably, some studies have even demonstrated that successful cognitive agers perform at a level commensurate with younger or middle-aged adults [8,9,10]. In a longitudinal study conducted by Lin et al. [11], 354 older adults were followed for 5 consecutive years, and their cognitive trajectories were examined using a mixed-growth model. Participants were stratified into three discrete categories: successful agers, those experiencing cognitive decline, and individuals with low, stable cognitive performance. Notably, the results unveiled the substantial impact of gender, apolipoprotein E (ApoE) ε4 status, and β-amyloid levels on the cognitive trajectories within all three groups. Another investigation, under the guidance of Halaschek-Wiener et al. [12], investigated a cohort of 480 “super seniors”, aged 85 to 105, who had no prior diagnoses of major health conditions. They were compared to a control group consisting of 545 middle-aged individuals aged 41 to 54. The outcomes delineated that the super-elderly cohort exhibited elevated levels of cognitive and functional abilities, superior physical functioning, and lower levels of depression in comparison to the control group. It is noteworthy that the super-elderly cohort demonstrated a reduced prevalence of smoking, while alcohol consumption frequency remained similar between the two groups. Moreover, Gefen et al. [9] defined the super-elderly as individuals aged 80 years or older who outperformed the average scores of those aged 56–66 years on cognitive tests. This specific group was longitudinally monitored, and no notable declines in neuropsychological measures encompassing memory, attention, language, or executive function were observed between baseline and an 18-month follow-up. This suggests that, collectively, they maintained consistent cognitive performance in both memory and non-memory domains.

In recent years, there has been a growing scholarly emphasis on scrutinizing the cerebral structural attributes and functional intricacies within cohorts that are characterized by exceptional cognitive resilience during the aging process. This scholarly pursuit leverages sophisticated neuroimaging methodologies to unravel the distinctive features associated with successful cognitive aging. Investigations into cerebral structure have revealed discernible patterns, wherein elderly individuals exhibiting remarkable cognitive aging showcase amplified cortical thickness or volume in specific neuroanatomical loci. This phenomenon is evident within the anterior cingulate cortex [8,13], as well as in specific regions of the default mode network, notably including the hippocampus [10] and the medial temporal lobe [14]. Longitudinal studies have been recognized as imperative for achieving a comprehensive grasp of the cerebral changes associated with successful cognitive aging. A recent study conducted by Marta et al. [15] incorporated cross-sectional and longitudinal analyses to scrutinize brain grey matter volumes in super-elderly individuals, compared with their age-matched elderly counterparts. The longitudinal cohort consisted of subjects aged 79.5 years or older who were distinguished by their cognitive success. The study comprised 64 super seniors (mean age 81.9 years, 59% female) and 55 age-matched regular seniors (mean age 82.4 years, 64% female). The cross-sectional analyses revealed that the super-elderly cohort exhibited elevated grey matter volumes in the medial temporal lobe, cholinergic forebrain, and motor thalamus, in contrast to their average elderly counterparts. Additionally, longitudinal analyses disclosed a decelerated rate of total brain grey matter atrophy in the super-elderly, particularly in the medial temporal lobe region. Dekhtyar et al. [16] conducted a study involving clinically normal adults participating in the Harvard Geriatric Brain Study, with cognitive testing and neuroimaging carried out at baseline and during a 3-year follow-up. The participants were categorized into two groups comprising best performers and typical performers, grouped according to their performance on challenging memory tests. The study revealed that the best performers exhibited heightened processing speed and executive function. Regarding brain structure, the best performers demonstrated a larger hippocampal volume at baseline, although no significant difference in amyloid load was observed. This study underscores the finding that the successful cognitive aging group excelled not only in cognitive performance but also exhibited distinctive brain structure characteristics. To augment our understanding further, it is imperative to explore alterations within the white matter. Chen et al. [17] conducted an extensive examination involving cognitively unimpaired older adults, specifically, 24 “SuperNormal” individuals and 24 cognitively normal controls, meticulously matched for age and education. Utilizing diffusion tensor imaging (DTI) scans at two time points separated by a two-year interval, the study conducted comparative analyses, unveiling significant connectivity alterations within diverse brain regions. These alterations spanned crucial areas such as the middle frontal, cingulate, parietal, temporal, and subcortical regions among the SuperNormals. In contrast to unimodal investigations, the imperative significance of multimodal research initiatives becomes evident in their capacity to yield a more comprehensive spectrum of information that is germane to the understanding of successful cognitive aging. In a scholarly pursuit by Baran et al. [18], a comprehensive investigation unfolded, integrating T1, amyloid PET, FDG-PET, and cognitive assessments over a meticulously recorded 6-month period. The investigation comprised a cohort of 172 individuals classified as cognitively normal, juxtaposed with 122 individuals manifesting successful cognitive aging. The latter cohort demonstrated a conserved state of cortical glucose metabolism, coupled with diminished levels of amyloidosis and glucose hypometabolism that were localized to the right isthmus cingulate cortex. In a study conducted by Sun et al. [10], brain MRIs were collected from 91 participants aged 18–35 years and from 47 participants aged 60–80 years. Superagers were defined as those individuals who met stringent psychometric criteria and achieved cognitive test results that were not below average for their age or even equal to or above the adjusted values for younger adults. Statistical analyses revealed that superagers exhibited a thicker cerebral cortex in key areas within both the default mode and salience networks, compared to typical older adults. Lastly, regarding episodic memory capacity, the successful cognitive aging population demonstrated comparable performance in middle-aged and older adults aged 50–65 years. However, imaging analyses indicated that the degree of brain atrophy in this population was not significantly different from that of middle-aged and older adults [13].

The above exposition underscores the substantial body of research dedicated to the scrutiny of the structural and functional cerebral attributes of individuals who experience successful cognitive aging, predominantly employing advanced brain imaging techniques. Concurrently, inquiries aimed at elucidating the characteristics of these cohorts through the lens of lifestyle practices have been diligently conducted. It is, however, paramount to recognize that many of these investigations have been encumbered by limitations, notably small sample sizes and a predominant focus on the assessment of grey matter volume and cortical thickness within the context of successful aging. This limitation accentuates the compelling need for more expansive sample sizes and the amalgamation of multiple imaging modalities, thereby facilitating a more all-encompassing exploration of the biomarkers underpinning the population who are experiencing successful cognitive aging. By adhering to this broader approach, researchers can plumb the depths of the intricate neural mechanisms that underscore successful cognitive aging, potentially unveiling novel associations between brain structure, lifestyle factors, and cognitive preservation in this elderly demographic. In this context, the current study adopts a multiparametric imaging approach, incorporating a T1-weighted MRI (T1), diffusion-weighted MRI (dMRI), and resting functional brain MRI (rs-fMRI), along with demographic variables, to glean deeper insights into the phenomenon of successful cognitive aging. This integrative methodology aims to provide a more nuanced understanding of the complex interplay between brain structure, function, and lifestyle attributes in older adults. In doing so, it endeavors to propel our comprehension of successful cognitive aging into a realm of greater comprehensiveness, thereby enriching our knowledge of this phenomenon.

## 2. Materials and Methods

### 2.1. Study Population

The UKB [19,20] represents a prospective, population-based study initiated in February 2006, encompassing more than 500,000 middle-aged and older volunteers aged between 40 and 69. The participants were recruited from 22 data collection sites situated across the United Kingdom. This endeavor captures a diverse array of data, including genetic sample information, multiparametric imaging data, lifestyle details, and biospecimen information. Ethical approval was obtained by the UKB from the Northwest Multi-Center Research Ethics Committee (REC reference 11/NW/0382, 31 May 2011). Approval for this particular study was granted by the UKB under application number 68382 (5 December 2021). Individuals exhibiting indications of brain diseases, as defined by the International Classification of Diseases ICD-10, were intentionally excluded from the analysis. Consequently, a total of 388,721 individuals were retained in alignment with this specific criterion.

### 2.2. Magnetic Resonance Imaging (MRI)

The UKB imaging [21] extension program has garnered participation from approximately 100,000 subjects and is scheduled to be completed by early 2023. The study utilized a diverse range of neuroimaging modalities, including a T1-weighted MRI (T1 MRI), diffusion-weighted MRI (dMRI), and resting-state functional MRI (rs-fMRI). Leveraging the capabilities of these modalities, valuable insights into the distinct aspects of brain structure and function were attained, facilitating a comprehensive investigation of the research objectives. Specifically, the T1 MRI provided detailed anatomical information, the dMRI allowed for the assessment of white matter connectivity, and the rs-fMRI captured intrinsic brain activity patterns. Further protocol details can be accessed at http://biobank.ctsu.ox.ac.uk/crystal/refer.cgi?id=2367, retrieved on 11 January 2022. All image data were acquired using a standardized German Siemens 3.0 T (manufactured by Siemens Healthineers, Erlangen, Germany) superconducting MRI scanner. Following data acquisition, a unified approach was employed for image pre-processing and preliminary analysis, culminating in the derivation of a comprehensive set of imaging-derived phenotypes (IDPs). For a comprehensive understanding of post-processing pipelines and data outputs, a detailed description is available at https://biobank.ctsu.ox.ac.uk/crystal/crystal/docs/brain_mri.pdf, accessed on 11 January 2022. These IDPs are meticulously designed to capture valuable insights into the different aspects of brain structure and function. Notably, as of late 2022, substantial progress had been achieved, with the UKB having published raw neuroimaging images and IDPs for over 40,000 individuals. Specifically, the T1 imaging data encompassed a total of 222 IDPs, representing various measurements and characteristics of brain tissue acquired through this imaging modality. The dMRI data, on the other hand, consisted of nine indices that capture the distinct properties of water molecule diffusion within the brain, resulting in a comprehensive dataset comprising a total of 675 IDPs. Furthermore, the rs-fMRI data encompassed 21 distinct independent components (ICs) that were representative of the resting-state brain’s network activity.

#### 2.2.1. T1-Weighted MRI (T1)

T1 MRI is a meticulously precise structural modality that has garnered recognition for its exceptional capacity to intricately capture brain anatomy at an impressive resolution. This imaging modality offers a potent contrast between grey and white matter, enabling the accurate visualization of brain structures. The quantification of volumes was executed through the utilization of the FMRIB software library (FSL, version 5.0.10), platform (http://fsl.fmrib.ox.ac.uk/fsl, accessed on 16 February 2022). Additionally, the FMRIB’s automated segmentation tool (FAST, version FAST3) was employed to derive a total of 139 IDPs [22], accomplished by summing the partial volume estimations within 139 regions of interest (ROIs). These ROIs, established in the MNI152 space, amalgamated parcellations from diverse atlases, including the Harvard–Oxford cortical and subcortical atlases (https://fsl.fmrib.ox.ac.uk/fsl/fslwiki/Atlases, accessed on 16 February 2022) and the Diedrichsen cerebellar atlas (http://www.diedrichsenlab.org/imaging/propatlas.htm, accessed on 16 February 2022). The previously estimated warp field, effectuating the transformation of subject data into a standardized space, was subsequently inverted and then applied to the ROIs, engendering a version of the ROIs in native space to achieve precise masking within the segmentation framework. For the segmentation of anatomical images and the generation of vertex meshes pertaining to specific subcortical structures, the Bayesian model-based segmentation toolbox, known as the FMRIB’s integrated registration and segmentation tool (FIRST, version 1.2), was employed [23]. This widely utilized software, available at (http://fsl.fmrib.ox.ac.uk/fsl/fslwiki/FIRST, accessed on 16 February 2022), facilitated the accurate delineation and quantification of 15 distinctive subcortical structures of interest. To account for individual variations in cranial size, the estimated volumetric measurements were subsequently subjected to normalization via a brain scale factor. In extracting the cortical thickness from cortical regions, the widely acknowledged FreeSurfer [24] parcellation scheme was judiciously implemented. This scheme, underpinned by the Desikan–Killiany atlas [25], furnishes a comprehensive delineation of cortical domains across both hemispheres, collectively encompassing a total of 68 discrete regions.

#### 2.2.2. Diffusion-Weighted MRI (dMRI)

The dMRI is a structural technique that can be employed to assess the movement of water molecules within the local tissue environment. By measuring water diffusion along various orientations, dMRI provides two types of derived phenotype variables. At the voxel level, local estimates of diffusion properties provide valuable insights into the integrity of microstructural tissue compartments, including diffusion tensor estimates. Conversely, long-range estimates derived from tractography, involving the tracing of brain pathways, offer valuable information about the structural connectivity between pairs of brain regions. For this study, the DTIFIT, available at (https://fsl.fmrib.ox.ac.uk/fsl/fdt, accessed on 16 February 2022), was employed to fit a diffusion tensor at each voxel, resulting in the generation of several diffusion measures. These measures include fractional anisotropy (FA), the tensor mode (MO), axial diffusivities (L1), radial diffusivities (L2, L3), and mean diffusivity (MD) maps, which collectively provide comprehensive insights into the characteristics of water diffusion within the brain tissue. Furthermore, the dMRI data underwent further processing using NODDI (neurite orientation dispersion and density imaging), a technique introduced by Mckee and Britton [26]. NODDI enables the estimation of white matter microstructural parameters, including the intracellular volume fraction (ICVF), isotropic water volume fraction (ISOVF), and the orientation dispersion index (OD). These parameters provide additional information regarding the microstructure of white matter and significantly contribute to a more comprehensive analysis of the relationship between cognitive function and white matter integrity.

To investigate white matter microstructure, tract-based spatial statistics (TBSS), a sophisticated analytical approach, was employed in this study [27]. TBSS facilitate the alignment of the FA image onto a standard-space white-matter skeleton using high-dimensional FNIRT-based warping [28]. This standardized-space warp is subsequently applied to all other DTI/NODDI measures. For each DTI/NODDI measure, the resulting skeletonized images were subjected to averaging across a set of 48 standard spatial tract masks, as defined by Susumi Mori’s group at Johns Hopkins University [29]. This meticulous averaging procedure yielded a total of 432 distinctive IDPs.

In addition to the TBSS analysis, a separate tractography-based analysis was conducted. This analysis commenced with the intra-voxel modeling of the multi-fiber bundle orientation structure using the BEDPOSTx tool (version 6.0.4) (a Bayesian estimation of the diffusion parameters obtained using sampling techniques). Subsequently, probabilistic tractography analysis was performed using PROBTRACKx (version 6.0.4) with cross-fiber modeling [30,31,32]. The output derived from BEDPOSTx generated bundle maps from seeding at either the voxel or region level. A standard ROI mask defined by AutoPtx was employed, which encompasses a set of 27 major bundle maps [28]. This process involved the computation of 9 dMRI metrics across each of the 27 brain regions, resulting in the extraction of 243 IDPs.

#### 2.2.3. Resting Functional Brain MRI (rs-fMRI)

The analysis of rs-fMRI images involved the implementation of the MELODIC (multivariate exploratory linear decomposition into independent components) framework [33]. This processing pipeline incorporated group principal component analysis and independent component analysis, leading to the extraction of spatially orthogonal independent components (ICs) that represented distinct resting-state neural networks [34]. To obtain a population-level spatial map of the resting-state network, a low-dimensional group ICA approach was implemented. The functional images were pre-processed using 25 fractions, with a rigorous exclusion process applied to eliminate 4 noise components. Consequently, a set of 21 components of particular interest was obtained [35] (Table 1). Each of these components corresponded to unique resting-state networks and provided invaluable insights into the underlying neural activity patterns during rest. The online visualization of these ICs is made possible through the Papaya viewer (https://www.fmrib.ox.ac.uk/ukbiobank/group_means/rfMRI_ICA_d25_good_nodes.html, accessed on 16 February 2022). The viewer and accompanying maps offer an interactive and insightful platform from which to explore and comprehend the spatial distribution of the ICs, derived from the resting-state functional magnetic resonance imaging (rs-fMRI) data.

### 2.3. Neuropsychological Testing

In this investigation, cognitive function was evaluated using a comprehensive battery of neuropsychological assessments (Table 2), which encompassed multiple tests designed to assess various cognitive domains [36]. These tests were carefully selected to assess different cognitive domains and provide insights into specific aspects of cognitive functioning. The administered tests included the trail-making test B, which serves as an indicator of executive function, and tower rearranging (the number attempted). Additionally, the matrix pattern completion task was employed to assess processing speed, specifically by measuring the duration needed by participants to answer each puzzle. The fluid intelligence of the participants was determined by calculating the sum of correct answers obtained for a specific task. Reaction time, reflecting the average time required to accurately identify matches in a task based on the “Snap” card game, was also included in the battery. Furthermore, the cognitive assessment encompassed pair-matching (the number of correct associations) to gauge visual memory, symbol digit substitution (the number of symbol digit matches made correctly) to evaluate processing speed, paired associate learning (the number of word pairs correctly associated) to measure verbal declarative memory, and numeric memory (the maximum number of digits recalled) as an index of working memory.

### 2.4. Definition of a Successful Cognitive Aging Population

The study retained a total of 17,072 subjects, with data from 3 imaging modalities (T1, dMRI, and rs-fMRI) and 9 cognitive tests. These subjects were divided into 2 groups based on a cut-off age of 65, resulting in a middle-aged group (*n* = 9050) and an older group (*n* = 8022). Principal component analysis (PCA) was employed as a dimensionality reduction technique to derive generic cognitive scores from a battery of nine cognitive tests. The scree plot analysis unveiled a conspicuous inflection point subsequent to the first principal component, elucidating a cumulative variance of approximately 30% within both demographic cohorts. Consequently, we exercised discretion in favor of retaining the first principal component, owing to its capacity to encapsulate the maximum variance. This strategic choice was consistently applied across both the older and middle-aged study cohorts. Subsequently, the subjects in the older group underwent two rounds of projection to obtain principal component scores corresponding to the first principal component. First, the principal component scores of the older age-group dataset were subjected to normalization through Z-score transformation, employing the dataset’s own mean and standard deviations. This procedure yielded the first set of generic cognitive scores. Only those subjects falling within the upper quartile of the score distribution (Z-scores > 0.67) were retained for further analysis. Continuing with the analysis, the principal component scores of the older age-group dataset were once again normalized using the Z-score transformation, this time based on the mean and standard deviation values of the middle-aged group. This generated a distinct set of generic cognitive scores. Subjects with scores exceeding 0 were retained in this instance. Subjects who did not meet the threshold criteria for either of the 2 groups were systematically excluded from the analysis, resulting in a cohort of 1986 subjects being retained for subsequent investigations. Finally, the process of standardizing individual cognitive scores involved a two-step procedure. Initially, this transformation relied upon the mean and standard deviation values specific to each cognitive score within the older age group. Subsequently, a second transformation was carried out, utilizing the mean and standard deviation values derived from the cognitive scores of the middle-aged group. For the remaining subjects, an additional layer of selection was applied through the implementation of specific score thresholds. These thresholds mandate that scores must exceed 0 during the initial transformation and surpass −0.67 during the subsequent transformation. Subjects who met these stringent threshold criteria across all four cognitive assessments (comprising the matrix, tower test, fluid IQ, and digital memory) were subsequently classified as constituents of the definitive cohort exemplifying successful cognitive aging. This rigorous and meticulous selection process culminated in the identification of a total of 1060 subjects. The final successful cognitive aging population consisted of 623 males and 437 females. Conversely, the remaining 6962 participants were categorized within the non-successful cognitive aging group.

### 2.5. Demographic Factors

Brain maintenance and cognitive maintenance are critical components of successful cognitive aging [37,38], encompassing the preservation of brain integrity and functionality over time and the proactive efforts to safeguard and optimize cognitive functions throughout the aging process. A prior study examined the intricate interplay between a comprehensive array of 16 demographic factors, including lifestyle choices and health status, and their impact on brain and cognitive maintenance. Drawing from this foundation, our present investigation focused on a subset of 12 indicators selectively retained from this previous study [39]. Specifically, these factors were classified into distinct categories to explore their relationship with the variables of interest. The smoking-related factors were further divided into two groups: smoking behavior (past smoking frequency, smoking status, and ever smoking) and smoking intensity (the number of pack-years smoked and the proportion of pack-years smoked relative to the hours smoked by adults). Additionally, three factors pertaining to alcohol consumption were analyzed, namely, drinking status, frequency of drinking, and ever drinking. To capture individuals’ daily activities, a diverse set of five indicators was employed, measuring various aspects of physical activity. These indicators included two-handed grip strength, usual walking speed, time spent watching TV, and time spent driving. Recognizing the significance of physical health, three factors were selected for analysis in relation to the potential impact on an individual’s quality of life: the presence of diabetes, diastolic blood pressure, and systolic blood pressure. By conducting a rigorous analysis of these selected demographic factors in relation to the attainment of successful cognitive aging, our study aims to achieve a more comprehensive understanding of their interdependence with successful cognitive aging.

### 2.6. Statistical Analyses

To investigate the potential differences between the successful cognitive aging group and the non-successful cognitive aging group, we utilized three imaging modalities. For normally distributed continuous variables, statistical analyses were performed using an analysis of variance (ANOVA) [40]. Meanwhile, non-normally distributed continuous variables were analyzed using two independent samples via Mann–Whitney U tests [41]. For nominal variables, chi-square tests were used [42].

In order to address the concerns regarding multiple comparisons that are inherent in the analysis of IDPs, we implemented a false discovery rate (FDR) threshold adjustment. This method served to mitigate the likelihood of Type I errors and enhance the rigor of our conclusions. A significance level of 0.01 was used, thereby ensuring conservative control over the probability of Type I error occurrence [43].

In order to ensure the robustness and validity of the IDP analysis, rigorous efforts were dedicated to addressing potential confounding factors [44]. One such confounder considered was the estimated intracranial volume, which was derived using the T1 scaling factor. This adjustment effectively normalizes brain size differences across individuals, thereby minimizing their potential impact on the analysis results. Furthermore, several covariates, including age, age squared, height, head movement, and scanner table position [45,46], were incorporated into the regression model. Subsequently, these covariates were carefully removed from the analysis to control for their potential influence on the observed effects. This meticulous process aimed to isolate and focus on specific cognitive factors of interest, enhancing the reliability of the IDP analysis.

## 3. Results

### 3.1. T1

**Figure 1:**
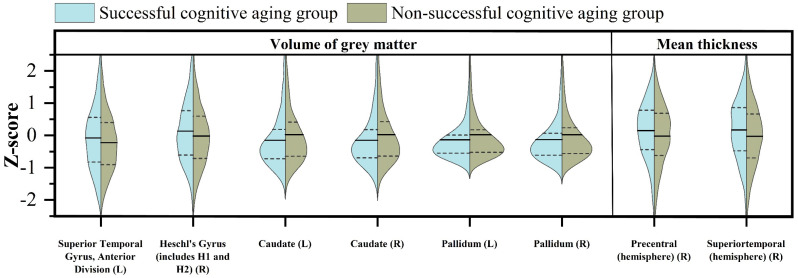
The brain regions wherein significant disparities in grey matter volume and cortical thickness were observed between a group characterized by successful cognitive aging and another group displaying non-successful cognitive aging (L, left; R, right).

Significant disparities in grey matter volume were observed between the two groups, highlighting the specific brain regions that contribute to successful cognitive aging (Figure 1). The left superior temporal gyrus, specifically its left anterior division, demonstrated notably higher grey matter volumes in the successful cognitive aging group (*p* = 0.001, f = 20.689). Similarly, the right Heschl’s gyrus exhibited significantly increased grey matter volumes in the two groups (*p* = 0.004, f = 21.333). Conversely, the non-successful cognitive aging group exhibited notably higher grey matter volumes in both the left and right caudate nucleus, as well as in the pallidum. Statistical analysis revealed significant differences (*p* < 0.001) for the caudate nucleus and for the pallidum (*p* < 0.001).

Regarding the mean thickness measurements, meaningful distinctions were detected in the precentral (right hemisphere) (*p* = 0.008, f = 25.896) and superior temporal region (right hemisphere) (*p* = 0.002, f = 36.274), with the successful cognitive aging group displaying higher mean thickness values. Subsequently, the brain regions exhibiting significant differences between the above two groups were utilized for further comparisons with the middle-aged group. These additional comparisons did not identify any regions that displayed statistically significant differences.

### 3.2. DMRI

**Figure 2:**
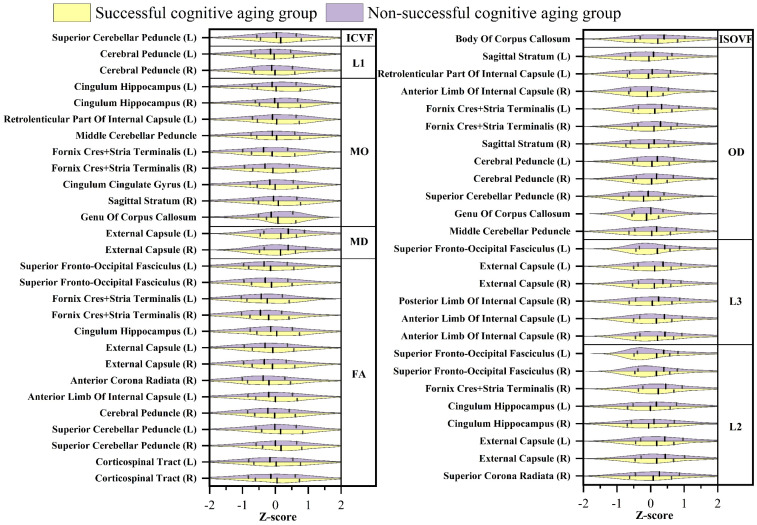
The white matter regions within the successful cognitive aging group that exhibit statistically significant deviations in diffusion MRI skeleton measurements compared to the non-successful cognitive aging group (L, left; R, right).

**Figure 3:**
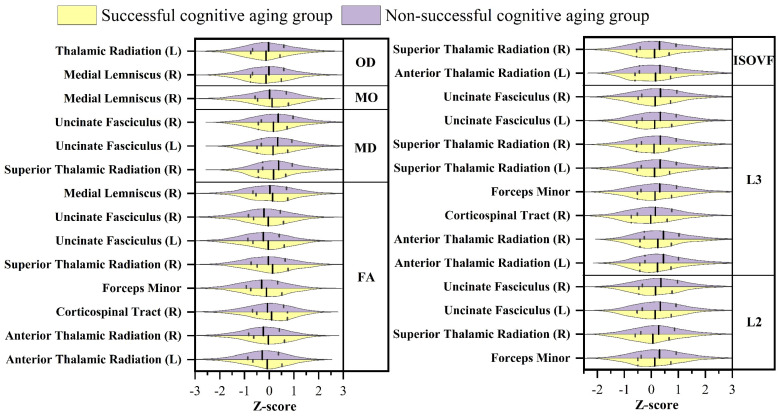
The white matter regions within the successful cognitive aging group that manifest noteworthy variations in diffusion MRI weighted means compared to the non-successful cognitive aging group (L, left; R, right).

In the comparative analysis of white matter microstructure, distinct patterns emerged when contrasting the successful cognitive aging group and the unsuccessful cognitive aging group. Specifically, the mean values of FA, MO, ICVF, and L1 were higher in the successful cognitive aging group compared to the unsuccessful cognitive aging group. Conversely, the mean values of MD, the L2 and L3 eigenvalues, OD, and ISOVF were lower in the successful cognitive aging group as opposed to the unsuccessful cognitive aging group. Further exploration of the dMRI skeleton measurements revealed 14 white matter regions wherein the 2 groups exhibited statistically significant differences in FA (*p* < 0.01) (Figure 2). Subsequently, the investigation revealed that the most notable disparities were observed in specific white matter regions, namely, the fornix crus+stria terminalis and the external capsule. These regions exhibited significant variations in FA, MD, L2, and L3 (*p* < 0.01). Among the dMRI weighted mean measures, FA and L3 demonstrated the highest number of significant differences, with a total of eight regions exhibiting statistically significant variations. Notably, no significant differences were observed in L1 and ICVF across any white matter regions (Figure 3). The white matter regions showing the most significance were in the superior thalamic region, which exhibited significant differences in FA, MD, L2, L3, and ISOVF (*p* < 0.01). These findings collectively indicate notable disparities in white matter microstructure between the successful cognitive aging and unsuccessful cognitive aging groups. The elevated FA, MO, ICVF, and L1 values, coupled with reduced MD, L2, L3, OD, and ISOVF values, in the successful cognitive aging group highlight the potential relevance of these microstructural metrics in the context of cognitive health and aging. Furthermore, a comparison was conducted on the white matter regions showing differences between successful and unsuccessful cognitive aging, to assess the significant differences in the white matter microstructure between the successful cognitive aging group and a middle-aged group. The findings revealed that approximately ten percent of the identified differential points exhibited no statistically significant variations between the successful cognitive aging group and the middle-aged cohort. These specific points of inconclusiveness encompassed variables such as the weighted mean FA value of the right corticospinal tract, the FA skeleton, the mean FA value of the left hippocampal sheath, the right hippocampal sheath L2 value, and the mean MO value of the geniculate portion of the corpus callosum, as well as the mean MO and OD values of the right sagittal lamina (*p* > 0.05).

### 3.3. Rs-fMRI

**Figure 4:**
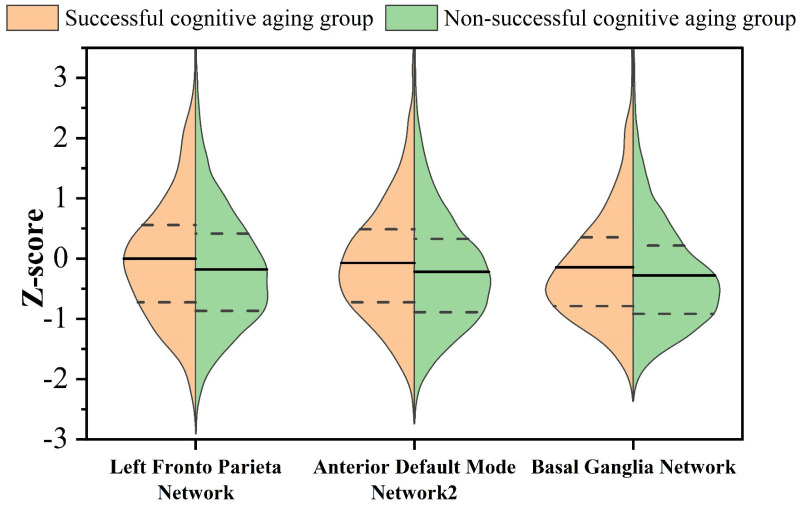
The ICs in which significant disparities in resting-state network activities were observed between a group characterized by successful cognitive aging and another group exhibiting non-successful cognitive aging.

In the context of rs-fMRI, the examination of successful and non-successful cognitive aging groups unveiled noteworthy distinctions in specific brain networks (Figure 4). Specifically, statistically significant differences were observed in the left frontoparietal network (*p* < 0.001, f = 30.966), the anterior default mode network 2 (*p* = 0.002, f = 21.783), and the basal ganglia network (*p* = 0.009, f = 19.589). These findings underscore the relevance of rs-fMRI in capturing the distinct patterns of functional activities within the brain that are associated with successful cognitive aging outcomes.

### 3.4. Demographic Analysis

In the successful cognitive aging group, the gender distribution indicated that 59% were male, while 41% were female. Furthermore, the age distribution within this group was as follows: 63.2% fell within the 65–70 age range, 31.4% were in the 70–75 age range, and a smaller proportion of 5% belonged to the 75–81 age range. Conversely, within the non-successful cognitive aging group, the gender distribution showed a more balanced distribution, with 51% being male and 49% female. The age distribution within this group varied slightly from that of the successful group, with 36.8% falling within the 65–70 age range, 43.4% in the 70–75 age range, and 14.6% belonging to the 75–82 age range. Regarding educational attainment, a notable disparity was observed between the two groups under investigation. Specifically, among the successful cognitive agers, an impressive 70 percent had obtained a college degree, which was in stark contrast to the non-successful cognitive aging group, where only 51 percent had achieved a similar level of educational attainment. Furthermore, it is noteworthy that three percent of the non-successful cognitive agers possessed only primary-school education, while no individuals with such credentials were identified within the successful cognitive aging group.

**Figure 5:**
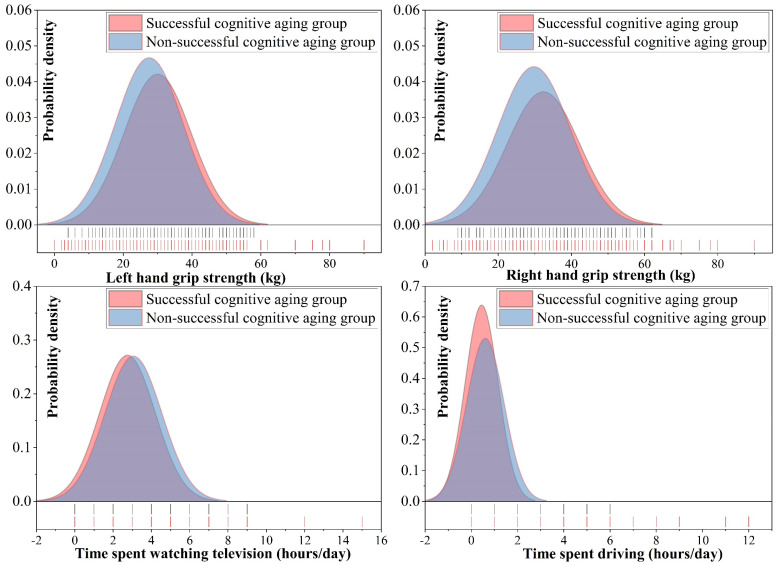
The probability density distributions of grip strength, time spent driving, and watching TV in relation to successful cognitive aging compared to non-successful cognitive aging groups.

**Figure 6:**
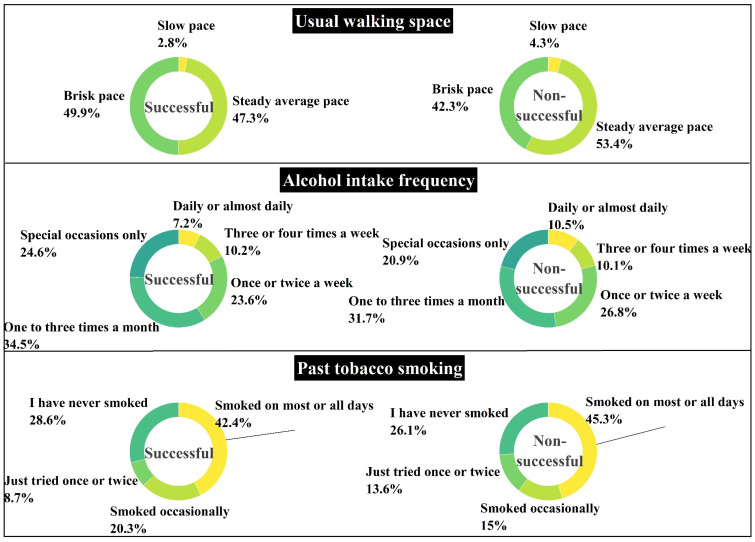
The frequency distributions of alcohol intake frequency, past tobacco smoking, and usual walking pace with respect to the successful cognitive aging versus non-successful cognitive aging groups.

Among the 12 indicators investigated, 7 of them exhibited statistically significant differences between the successful and non-successful cognitive aging groups. Notably, the two-handed grip strength criterion demonstrated a significant difference, with higher mean values of two-handed grip strength in the successful cognitive aging group (p_L = 2.14 × 10^−12^, f = 54.422; p_R = 1.45 × 10^−13^, and f = 59.764). In the domains of lifestyle and environment, there was also a significant distinction in the frequency of alcohol consumption (*p* = 0.002, χ^2^ = 24.712) (Figure 5. Additionally, smoking behavior exhibited significant differences between the two groups, with past tobacco smoking showing notable variations (*p* = 3.83 × 10^−7^, χ^2^ = 37.749) (Figure 6). Furthermore, significant differences were observed in physical activities, specifically in terms of the usual walking pace (*p* = 1.2 × 10^−5^, χ^2^ = 15.238), time spent watching TV (*p* = 7.32 × 10^−8)^, f = 32.807) and time spent driving (*p* = 3.03 × 10^−10^, f = 44.851) (Figure 6). These findings highlight the relevance of these lifestyle and physical activity indicators in the context of successful cognitive aging outcomes.

## 4. Discussion

This study represents a substantial advancement in our understanding of successful cognitive aging, a phenomenon that may be intricately linked to beneficial lifestyle practices that empower individuals to manifest elevated cognitive capabilities. Simultaneously, those who achieve successful cognitive aging seem to enjoy heightened safeguards within those brain regions closely linked to diverse cognitive functions. Our rigorous comparison and analysis of the neuroanatomy of individuals in the successful cognitive aging group relative to those in the non-successful cognitive aging group have unveiled notable disparities in the activity of resting-state networks, as well as the structural attributes of both grey and white matter. These findings offer invaluable insights into the multifaceted interplay between brain function, structure, and lifestyle choices in the context of cognitive aging.

### 4.1. Grey Matter Morphology

In our investigation of grey matter structure within those groups characterized by successful cognitive aging, we have made noteworthy observations suggesting that individuals exhibiting successful cognitive aging may demonstrate enhanced auditory perception and voluntary motor control in comparison to their counterparts with non-successful aging. Firstly, we identified substantial differences in the regions of the superior temporal gyrus and Heschl’s gyrus between the two groups. These regions play a pivotal role in auditory perception [47], with Heschl’s gyrus specifically playing a central role in pitch intuition within the superior temporal cortex [48]. Given the significance of these identified brain regions in auditory processing, it is essential to underscore their potential relevance in the context of successful cognitive aging. Notably, subclinical hearing impairment has been independently associated with declining cognitive function in older adults [49]. Notably, a report has identified hearing loss as a significant risk factor for cognitive decline, with a substantial portion of dementia cases attributable to mid-life hearing impairment [50]. The prevalence of hearing loss in the elderly population and its potential impact on cognitive function have been underscored in studies by Xu et al. [51] and Pichora Fuller et al. [52]. In light of this evidence, the preservation of brain regions associated with successful cognitive aging among elderly individuals gains importance in the context of auditory function. Furthermore, our findings revealed thicker cortical thickness in the precentral gyrus of successful cognitive agers compared to non-successful cognitive agers. The precentral gyrus is the primary motor cortex and is involved in voluntary movements [53]. Previous studies have shown that successful cognitive agers excel in the control of voluntary movement [54]; training for such control has been linked to slowing cognitive decline and preventing dementia in older adults [55,56]. This is further substantiated by our observations of demographic disparities between the two groups, with successful cognitive agers displaying higher grip strength and swifter walking speeds. Interestingly, we unexpectedly observed higher grey matter volume in the left and right caudate nuclei and pallidum in the non-successful cognitive aging group. It is essential to consider the potential limitations of subcortical nuclei segmentation using tissue segmentation tools, as their accuracy may not be as precise as the segmentation of cortical grey matter [57,58]. Additionally, previous research has reported varying associations between subcortical nuclei volumes and other factors. For instance, one study [59] found a positive association between white matter hyperintensity (WMH) load and caudate volumes in participants from the UKB. Studies investigating larger age ranges have reported a U-shaped curve for caudate volumes, which decrease from early adulthood to the 60s age range and then increase afterward [60,61]. Given that WMHs generally occur within the same age range and are more frequently observed in individuals experiencing non-successful cognitive aging, it is plausible that the elevated grey matter volume found in the caudate nuclei and pallidum of the non-successful cognitive aging group may be attributed to segmentation errors arising from the presence of WMHs in this specific population.

### 4.2. Brain Network Connectivity Strength

The intricate network architecture of the human brain has garnered increasing attention within the neuroscientific community for its potential to shed light on diverse aspects of human cognition, development, aging, and the repercussions of disease or injury [60,62]. Studies focusing on individuals experiencing successful cognitive aging have consistently highlighted their tendency to manifest greater preservation of cortical thickness or volume in the pivotal regions of specific brain networks, notably, the default mode network and salience network [8,9,10,13]. In alignment with these findings, our study has yielded comparable results, revealing that the group characterized by successful cognitive aging demonstrated superior network activities in the frontoparietal network, anterior default mode network, and basal ganglia network when compared to their non-successful cognitive aging counterparts. Among these networks, the frontoparietal network assumes a pivotal role in mediating the interplay between executive control and conscious perception, showing a robust correlation with attentional processes [63]. Studies have illustrated that older adults with higher levels of education exhibit heightened activation within the left frontoparietal network and, consequently, demonstrate enhanced attentional control [64]. Conversely, the basal ganglia network assumes significant importance in executive control, as it governs motor functions, emotional regulation, and various other vital processes. Dysfunction within the basal ganglia network is a prominent factor in the onset of conditions such as Parkinson’s disease [65]. Notably, the functional integrity of the executive control network has been linked to the preservation of executive abilities in individuals with mild cognitive impairment [66]. In addition to the fronto-parietal and basal ganglia networks, the default mode networks have received significant attention in the context of resting-state networks. These networks are most active during periods of rest and have been associated with human thought processes. Specifically, the anterior default mode network is particularly relevant to thought activity [67]. Taken together, these findings collectively suggest that individuals experiencing successful cognitive aging may present augmented executive functioning, memory retention, emotional regulation, and an array of cognitive proficiencies in their daily lives [68].

### 4.3. White Matter Microstructure

Previous studies investigating age-related changes in white matter integrity using dMRI have consistently demonstrated a linear decline in FA throughout the adult lifespan, while MD exhibits a quadratic rise from approximately 60 years of age [69,70,71]. Furthermore, investigations into FA, ICVF, and OD have revealed lower values in older age groups, whereas MD, ISOVF, and L2 and L3 exhibit higher values in such groups [72,73]. In our study, we observed that the mean values of FA, MO, ICVF, and L1 were higher in the successful cognitive aging group compared to the non-successful cognitive aging group, whereas the mean values of the other indicators were lower in the successful cognitive aging group. Particularly noteworthy is the sensitivity of FA, which exhibited differences in 14 white matter regions on FA skeleton measurements, making it the most informative parameter for distinguishing between the two groups. Subsequently, MO and L3, when assessed in tract analysis, demonstrated significant differences in eight brain regions. Conversely, L1 and ICVF did not appear to be as sensitive in detecting differences between the successful and non-successful cognitive aging groups. Quantitative studies focusing on FA, MO, and L3 as measures of white matter integrity can be instrumental in enhancing our understanding of the pathophysiological aspects underlying the cognitive changes associated with successful cognitive aging. In our study, we observed that the external capsule exhibited significantly higher values in the successful cognitive aging group compared to the non-successful cognitive aging group across parameters, including FA, MD, L2, and L3; the fornix cres+stria terminalis exhibited significantly higher values parameters, including FA, MO, L2, and OD. These findings align with previous studies that have reported age-related declines in white matter integrity, wherein the fornix, external capsule, and corpus callosum have been identified as the regions most affected by age-related changes [74,75]. Bennett et al. [74] highlighted the fornix and external capsule as regions demonstrating the most significant decline in white matter integrity with age. This suggests that these specific regions may play a crucial role in age-related cognitive changes. Additionally, studies have found that higher cognitive performance may be associated with the integrity of specific white matter tracts, such as the left superior and inferior longitudinal fasciculi, left fornix, and corpus callosum [76]. In our study, the right superior thalamic radiation emerged as the region with the most significant differences in various parameters, including FA, MD, L2, L3, and ISOVF. Notably, the thalamus has been implicated in microstructural degradation during normal aging, which is associated with cognitive decline, particularly in the contexts of attention, situational memory capacity, and information processing [77]. In contrast, our findings suggest that successful cognitive agers may retain relatively intact white matter structures in the superior thalamus, which could potentially contribute to their enhanced performance in memory and information-processing tasks. The preservation of white matter integrity in this region may be a key factor in supporting cognitive functions in successful cognitive aging. These brain regions, including the thalamus, have also been the focus of research in neurodegenerative diseases. In studies of mild cognitive impairment, reduced connectivity in the medial temporal lobe cortex and reduced white matter integrity in regions such as the cingulate gyrus and fornix were associated with cognitive impairment [78]. Similarly, in Parkinson’s disease, reduced FA values and higher eigenvalues (L2 and L3) were observed in various white matter tracts, including the corticospinal tract, corpus callosum, internal and external capsule, corona radiata, thalamic radiations, sagittal fasciculus, cingulate gyrus, and superior longitudinal fasciculus [79]. In the context of successful cognitive aging, the observed greater differences in white matter integrity compared to grey matter atrophy and the changes in resting state networks can be attributed to several fundamental factors. Firstly, the integrity of white matter serves as a foundational element for brain reserve [80], facilitating efficient communication and connectivity between diverse brain regions [81]. With advancing age, the degradation of white matter tracts disrupts the synchronized transmission of neural signals, leading to compromised cognitive function. However, in successful cognitive agers, the preservation of white matter integrity enables the uninterrupted flow of information across brain networks, facilitating the seamless coordination of cognitive processes. Secondly, a critical aspect contributing to the prominence of white matter integrity in successful cognitive aging is its role in bolstering cognitive reserve. Cognitive reserve [82], referring to the brain’s adaptive capacity to compensate for age-related changes or injuries, allows individuals to sustain cognitive function despite potential damage. Remarkably, scientific investigations have established a significant association between higher white matter integrity and greater cognitive reserve, highlighting the structural basis that white matter provides for cognitive resilience [83]. Consequently, even in the presence of age-related grey matter atrophy or network changes, individuals with preserved white matter integrity can effectively harness cognitive reserve to maintain their cognitive performance. Thirdly, white matter tracts play a pivotal role in establishing long-range connectivity between disparate brain regions, thereby facilitating the integration of specialized functions into complex cognitive tasks [84]. The maintenance of white matter integrity fosters the efficient transfer of information across distant brain regions, which is crucial for supporting higher-order cognitive processes such as working memory, problem-solving, and attentional control. Preserving white matter integrity emerges as a compelling avenue for interventions aimed at promoting successful cognitive aging and ameliorating age-related cognitive decline.

### 4.4. Factors of Life

In the course of a comparative analysis concerning the demographically relevant attributes of individuals experiencing successful cognitive aging and those facing non-successful cognitive aging, noteworthy distinctions emerged, centering primarily on lifestyle factors. In the realm of comprehending the intricacies of successful aging, lifestyle indicators assume a paramount role. Among these indicators, seven stand out as particularly valuable metrics. Within the purview of healthcare, these metrics not only contribute to our comprehension of the determinants of cognitive health but also provide actionable insights for the development of interventions aimed at promoting and sustaining cognitive well-being in the aging population.

The neurological ramifications of smoking and its deleterious impact on cognitive function have been extensively investigated and are now firmly established [85]. Numerous studies consistently demonstrate a correlation between smoking and a decline in cognitive abilities, including attention, memory, and executive function [86,87,88]. Furthermore, smoking has been associated with structural alterations in the brain, characterized by reduced grey matter volume and diminished thickness in those regions most closely linked to cognitive processing [89]. These detrimental effects can be attributed to the toxic constituents present in tobacco smoke, such as nicotine and carbon monoxide, both of which can inflict damage on the brain and impede cognitive processing [88]. In alignment with these established findings, our investigation explored the association between smoking history and cognitive aging outcomes. Notably, the proportion of individuals in the non-successful aging group who reported smoking most of the time was 2.5% higher compared to those in the successful cognitive aging group. Conversely, the proportion of never-smokers was 3.8% higher in the successful cognitive aging group compared to their non-successful counterparts. Smoking, and nicotine in particular, has a profound impact on various cognitive domains [90]. Consequently, the well-documented adverse effects of smoking on cognitive function likely contribute to the observed association between smoking and successful cognitive aging.

The excessive consumption of alcohol is a prevailing public health concern with potential implications for brain health and cognitive function. A considerable body of research has established links between alcohol intake, structural brain changes, and cognitive deficits, particularly in cases of heavy and prolonged drinking [91,92]. Our findings revealed that the non-successful cognitive aging group demonstrated a higher frequency of daily or almost daily drinking, with a 2.9% increase compared to the successful cognitive aging group. Similarly, the percentage of the non-successful cognitive group who consumed alcohol 3 to 4 times per week was 0.2% higher than their successful cognitive aging counterparts. Intriguingly, the successful cognitive aging group was identified as having a higher percentage of individuals who drank alcohol 1–3 times per month (3.2% higher) and only on special occasions (3.9% higher) compared to the non-successful cognitive aging group. These observations suggest that the drinking habits of the successful cognitive aging group may reflect a more moderate and sensible approach toward alcohol consumption. This aligns with prior research indicating the potential cognitive performance benefits associated with moderate alcohol intake [93]. However, it is imperative to acknowledge that the relationship between alcohol consumption and cognitive functioning is non-linear. Studies, such as that by Piumatti et al. [94], based on data from the UKB have demonstrated that while cognitive performance may initially improve with increasing alcohol consumption, it eventually declines when beyond a certain threshold. The observed association between moderate drinking habits and cognitive success underscores the importance of adopting responsible alcohol consumption practices among older adults to potentially support cognitive health.

Engaging in physical activities and sports offers a plethora of advantages for brain health and cognitive function, rendering them integral components of a health-promoting lifestyle [95,96]. The evaluation of diverse facets of physical activity encompasses five metrics, including bilateral grip strength, walking pace, driving time, and TV-watching duration. Significantly, grip strength, in particular, stands as a widely recognized and extensively validated metric for gauging overall health status [97], one that is increasingly acknowledged for its potential as a marker for psychiatric vulnerability and neurodegeneration in older adults [98,99]. In our investigation, we found compelling evidence of a significant difference in grip strength between individuals experiencing successful cognitive aging and those facing non-successful cognitive aging. Specifically, the successful cognitive aging group demonstrated notably higher grip strength, with a mean difference of 2.4 kg in the left hand and 2.6 kg in the right hand. These findings are consistent with previous research indicating that higher grip strength may be indicative of better motor coordination and control. Several studies, including those conducted by Seidler et al. [100], Gonzalez et al. [101], and Chou et al. [102], have reported a positive correlation between grip strength and superior cognitive and executive functions in individuals. Further supporting these observations, a cross-sectional analysis conducted by Jiang et al. [103], involving data from the UKB, demonstrated that greater grip strength was linked to improved cognitive functioning, heightened life satisfaction, increased subjective well-being, and reduced symptoms of depression and anxiety. Additionally, their results revealed widespread associations between stronger grip strength and increased grey matter volume, particularly in the subcortical regions and temporal cortices of the brain. These neuroanatomical associations suggest potential underlying mechanisms that may contribute to the observed cognitive benefits associated with greater grip strength. Accidental falls and the resulting injuries pose a significant threat to individuals aged 65 and above, with falls being the primary cause of accidental deaths in this age group [104]. Importantly, a considerable proportion of these falls occur during locomotion, highlighting the criticality of addressing issues related to walking and movement in older adults [105]. Our study revealed that successful cognitive agers exhibited a 7.6% higher percentage regarding walking at a brisk pace compared to the non-successful cognitive aging group. Concurrently, the percentage of slow walkers was 1.5% lower. Physical limitations, such as muscle weakness and loss of flexibility, have been implicated as significant factors contributing to the decline in walking speed observed in older adults. Muscle weakness, particularly in the lower extremities, can lead to compromised force generation during walking, resulting in a slower gait pattern [106]. Similarly, a loss of joint flexibility, notably in the hips, knees, and ankles, may restrict the range of motion in the gait, contributing to a more cautious and constrained walking style [107]. Importantly, research has established a strong correlation between decreased walking speed and cognitive decline in older adults. Slower walking speeds have also been identified as potential predictors of dementia-related diseases in this population [102,108]. These results highlight the potential of walking speed as an indicator of cognitive status in the aging population. In our brain structure findings, successful cognitive agers demonstrated a thicker cortex in the precentral gyrus, which is strongly linked to motor control, compared to non-successful cognitive agers [53]. This observation may contribute to the significant differences in grip strength and walking speed observed between successful cognitive agers and their non-successful counterparts. Unsuccessful lifestyles, such as sedentary behavior, limited physical exercise, and prolonged inactivity, have been consistently linked to cognitive decline and an elevated risk of chronic diseases. In this study, we observed a notable correlation, indicating that the successful cognitive aging group engaged in 0.5 h less daily television viewing and 2 h less daily driving compared to individuals experiencing non-successful cognitive aging. These findings intriguingly suggest that reduced participation in sedentary activities, such as limiting television viewing and driving duration, may potentially contribute to more favorable cognitive aging outcomes. Importantly, a sedentary lifestyle has been robustly associated with unsuccessful brain aging and cognitive decline. At the cellular level, sedentary behavior is characterized by significant changes, including astrocyte hypertrophy, myelin dysregulation, neurovascular dysfunction [109], and impaired neurogenesis [110]. These cellular alterations are believed to contribute to the observed decline in cognitive function and overall brain health among individuals leading a sedentary lifestyle.

### 4.5. Limitations

Our study grapples with multifaceted limitations, primarily originating from the absence of a universally accepted definition for successful cognitive aging. The definitions of success vary among researchers, being shaped by their distinct research objectives. In this study, we operationalize successful cognitive aging as a demographic with enhanced cognitive function across multiple domains that closely mirrors middle-aged individuals. This inclusive approach aims for a comprehensive exploration. However, acknowledging the variability in definitions is crucial since this introduces subjectivity and potentially impedes comparability with other investigations. Another notable limitation revolves around the delineation of 65 years as the threshold distinguishing middle age from old age, a construct that is deeply entrenched in historical, social, and demographic contexts. However, contemporary societal dynamics present a paradigm shift, with individuals aged 65 and beyond actively participating in full-time employment, travel, social interactions, physical fitness pursuits, and experiential spending. This evolving landscape suggests that the threshold age of 65 may no longer precisely align with contemporary societal norms. Additionally, we acknowledge the temporal limitations inherent in our cross-sectional study design. This precludes our ability to capture cognitive changes in the subjects across different time intervals. Consequently, we cannot discern the trajectory of cognitive aging or elucidate the dynamic nature of cognitive abilities over time. The intricacies of brain network dynamics present another challenge, wherein reported functional outcomes are solely derived from static network analyses, limiting our understanding of dynamic neural interactions. To surmount this issue, future research should broaden the analytical framework to explore inter-network connectivity and dynamic connectivity dynamics, fostering a more comprehensive understanding of brain activity. In the assessment of physical activity metrics, limitations arise from the indirect nature of some measures. Despite recognizing the importance of these measures, their indirect nature prompts us to advocate for future research to consider the integration of more direct measures to enhance precision and accuracy in gauging physical activity. Acknowledging the potential heterogeneity within the aging processes [111,112], treating successful cognitive aging as homogeneous may overlook critical variations. Consequently, in future research, it will be vital to employ brain imaging analysis methods that account for this heterogeneity [113], fostering a more nuanced understanding of the intricate neural mechanisms underlying successful cognitive aging. Lastly, we employed IDPs obtained from the UKB dataset, which were processed using various widely utilized neuroimaging software packages. These software applications have garnered extensive usage in a multitude of neuroimaging investigations. However, it is important to recognize that achieving optimal results often necessitates parameter adjustments tailored to the specific requirements of different imaging studies. In the context of processing vast datasets through complete automation, it is crucial to acknowledge that the generation of IDPs may not be guaranteed to be entirely free from inaccuracies or bias. While these software packages are valuable tools, the potential for processing-related biases should be considered when interpreting the results.

## 5. Conclusions

In conclusion, this study represents a significant advancement in our understanding of successful cognitive aging. Successful cognitive agers exhibit heightened safeguards in brain regions that are linked to diverse cognitive functions, emphasizing the interplay between neuroanatomy, lifestyle choices, and cognitive aging. Grey matter morphology analysis highlights thicker cortical regions in those areas associated with auditory perception and voluntary motor control functions in successful cognitive agers. Network connectivity strength analysis reveals superior activities in those critical brain networks related to executive control, memory retention, and emotional regulation in successful cognitive agers. Remarkably, our brain imaging analysis unveils a revelation: individuals who undergo successful cognitive aging exhibit a higher degree of integrity in their white matter microstructure. This noteworthy observation not only underscores the potential significance of white matter microstructure as a more sensitive discriminator of successful cognitive aging but also underscores the imperative need for future investigations that place a heightened emphasis on probing alterations in white matter microstructure. Numerous factors of life, including smoking, alcohol consumption, physical activity, and sedentary behavior, exhibit notable associations with cognitive aging outcomes. Such inquiries promise to offer profound insights into the underlying mechanisms governing cognitive preservation and successful aging. This innovative dimension of our research holds immense promise in furthering our comprehension of the intricate interplay between lifestyle factors, cerebral structure, and the process of cognitive aging. In the long run, it has the potential to pave the way for the development of precisely tailored interventions that are designed to bolster and sustain successful cognitive aging within the aging population. In essence, this research underscores the profound implications for cognitive aging, offering a potential path toward an improved quality of life and a brighter future for elderly individuals.

## Figures and Tables

**Table 1:** The 21 brain network correspondence ICs, along with their corresponding brain networks. These ICs represent different resting-state neural networks. Each IC is associated with a specific brain network, as outlined in the table.

IC	Resting-State Network	IC	Resting-State Network
1	Anterior default mode network	12	Sensorimotor network 3
2	Lateral visual network	13	Left cingulo-opercular network
3	Sensorimotor network	14	Anterior default mode network 2
4	Medial visual network	15	Cerebellar network
5	Right frontoparietal network	16	Executive control network
6	Left frontoparietal network	17	Auditory network 2
7	Posterior default mode network 2	18	Basal ganglia network
8	Medial visual network 2	19	Lateral visual network 2
9	Posterior default mode network	20	Precuneus/pcc default mode network
10	Sensorimotor network 2	21	Right cingulo-opercular network
11	Auditory network		

**Table 2:** Cognitive domains, neuropsychological tests, and test descriptions.

Testing	Description	Cognitive Domain	UKB ID
Pair-matching	Number of incorrect matches made in round	Visual declarative memory	399
Numeric memory	Maximum number of digits remembered correctly	Working memory	4282
Fluid intelligence	Fluid intelligence score assessment	Verbal and numerical reasoning	20016
Paired associate learning	Number of correctly associated word pairs	Verbal declarative memory	20197
Matrix pattern completion	Number of correctly solved puzzles	Non-verbal reasoning	6373
Reaction time	Mean time taken to correctly identify matches	Processing speed	20023
Symbol digit substitution	Number of correct symbol digit matches made	Processing speed	23324
Tower rearranging	Number of correctly solved puzzles	Executive function	21004
Trail-making	Duration needed to complete an alphanumeric path	Executive function	6350

## Data Availability

The imaging datasets generated by the UK Biobank analyzed during the current study are available via the UK Biobank data access process (see http://www.ukbiobank.ac.uk/register-apply/, accessed on 11 January 2021). The UK Biobank’s Research Access Administration Team handles all data access requests from academic and commercial researchers without any preference or exclusivity. The requests are evaluated based on whether they support health research in the public interest, and, if so, they are approved rapidly. Detailed information about the data available from the UK Biobank is available at http://www.ukbiobank.ac.uk, accessed on 11 January 2021. The exact number of participants with imaging data currently available from the UK Biobank may differ slightly from those described in this paper.
